# Endothelial Function and Serum Concentration of Toxic Metals in Frequent Consumers of Fish

**DOI:** 10.1371/journal.pone.0112478

**Published:** 2014-11-17

**Authors:** Silvio Buscemi, Sonya Vasto, Francesca Di Gaudio, Giuseppe Grosso, Sonia Bergante, Fabio Galvano, Fatima Maria Massenti, Emanuele Amodio, Giuseppe Rosafio, Salvatore Verga

**Affiliations:** 1 Dipartimento Biomedico di Medicina Interna e Specialistica Laboratorio di Nutrizione Clinica, University of Palermo, Palermo, Italy; 2 Dipartimento di Scienze e Tecnologie Biologiche, Chimiche e Farmaceutiche, University of Palermo, Palermo, Italy; 3 Dipartimento di Scienze per la Promozione della Salute e Materno Infantile, University of Palermo, Palermo, Italy; 4 Dipartimento di Scienze del Farmaco, University of Catania, Catania, Italy; 5 Scientific Institute for Research, Hospitalization and Health Care, Policlinico San Donato, San Donato, Milan, Italy; University of Pecs Medical School, Hungary

## Abstract

**Background:**

Endothelial dysfunction is involved in the pathogenesis of atherosclerosis. Consumption of fish is associated with reduced cardiovascular risk, but there is paucity of data concerning its effect on endothelial function. Furthermore, investigation of the effects of fish consumption on health must take into account the ingestion of contaminants, including transition metals and some metalloids, which may have unfavorable effects on health, including those on the cardiovascular system. We investigated the association between fish consumption, endothelial function (flow mediated dilation of the brachial artery), and serum concentration of some toxic metals in apparently healthy people.

**Methods:**

Twenty-nine high fish consumers (at least 3 portions a week) were compared with 25 low fish consumers (less than 1 portion a week). All participants were free of diabetes, cardiovascular or other systemic diseases. Serum metal (antimonium, arsenic, mercury, lead, cobalt, copper, zinc, selenium, strontium) concentrations were measured in subgroups of 24 high fish consumers and 19 low fish consumers.

**Results:**

Both groups exhibited similar habitual dietary patterns, age and anthropometric characteristics. The high fish consumers had higher flow mediated dilation (9.7±1.8 vs. 7.3±1.9%; P<0.001), but also higher serum concentrations of mercury (5.87±2.69 vs. 1.65±1.10 mcg/L; P<0.001) and arsenic (6.04±3.25 vs. 2.30±1.58 mcg/L; P<0.001). The fasting plasma glucose concentrations were significantly correlated with both mercury (r = 0.39; P = 0.01) and arsenic concentrations (r = 0.55; P<0.001).

**Conclusions:**

Habitual consumption of high amounts of fish is associated with better endothelial function despite higher serum concentrations of mercury and arsenic.

## Introduction

Atherosclerotic cardiovascular disease is the leading cause of morbidity and mortality in the Western world [1]. Both inflammation and endothelial function play a key role in the activation and progression of atherosclerosis [2]. In particular, increased production of reactive oxygen species (ROS), as well as oxidative and inflammatory stresses, are associated with impaired endothelial function. A reduced bioavailability of nitric oxide, a potent vasodilator and inhibitor of platelet adhesion and aggregation, with anti-inflammatory and anti-proliferative properties [3], [4], is the hallmark of endothelial dysfunction. Endothelial function can be measured in vivo by flow mediated dilation (FMD) in the brachial artery, which has proven to be a strong predictor of cardiovascular events [5]–[9]. Endothelial function is influenced by many factors, including insulin resistance, diabetes, dyslipidemia, drugs, diet and some foods [10]–[15]. Data have shown that fish consumption should be part of a healthy diet, and helps reduce the risk of cardiovascular diseases [16]–[18]. We recently found that consuming fish at least 2 times a week is associated with less carotid atherosclerosis in apparently healthy people [19]. Fish consumption influences the pathways leading to atherosclerosis in several ways. n-3 polyunsaturated fatty acids (n-3 PUFAs) have been indicated as the most active component of fish in mediating cardiovascular protection. Fish consumption is associated with decreased circulating biomarkers of endothelial dysfunction and inflammation [20], and FMD values seem to improve after n-3 PUFA intake [21]. However, there is paucity of data concerning the association between levels of habitual fish consumption and FMD, and it is unknown what the level of habitual fish intake that influences the FMD is. Furthermore, the relationship between fish intake and health is even more complex considering that fish contaminants, such as metals and other toxic substances, have been recently associated with adverse effects on the cardiovascular system [22]–[26], diabetes [27]–[29] and tumors [30].

In this study we investigated whether regular fish consumption is associated with improved endothelial function, measured by FMD. We also determined blood concentrations of metal toxic contaminants, such as mercury and arsenic, in relation to the level of fish consumption.

## Methods

### Participants

The participants in this study were recruited among those who took part in a previous study [31], which investigated the association between dietary patterns, carotid atherosclerosis, and insulin resistance in 1,231 participants (465 males and 766 females). In that study, semi-quantitative habitual intakes of different foods during the preceding 12 months were assessed with the Food Frequency Questionnaire (FFQ) [32]. A retrospective, cluster analysis of the same cohort was conducted to identify dietary patterns, a procedure that is based on the intercorrelation among food groups or nutrients. A diet that could be defined as Unhealthy was identified: this was characterized by high consumption of soft drinks, fried foods, seed oils, cured meats, butter, red meat and sweets; a dietary pattern that resembled the Mediterranean diet, defined as healthy, was characterized by high intakes of fruit, milk and cheese, olive oil, vegetables, pasta and bread; a third pattern of dietary habits was defined as Intermediate, and had characteristics that lay between the two other diets. In our study, we examined low fish consumers (LFC), defined as those individuals who typically consumed less than 1 portion of fish per week, and high fish consumers (HFC) who consumed at least 3 portions of fish per week. People with any form of diabetes, cardiovascular or systemic disease, and women who were pregnant or lactating in the past 6 months, were excluded from this analysis. Exclusion criteria were also the use of acetyl-salicylic acid or other antiplatelet drugs, statins or fibrates, oral hypoglycemic drugs, nitrates, non-steroidal anti-inflammatory drugs, corticosteroids, drugs interfering with coagulation, supplementation with vitamins and/or anti-oxidants, and regular sports activity. There was no incentive provided to the participants. Our hospital’s Ethics Committee approved the study protocol, and each participant signed an approved informed consent form.

All participants were examined in the morning, and in post-absorptive fasting condition. A fasting blood sample was obtained for biochemical measurements and serum samples were frozen at −80°C for subsequent analysis. Height, body weight and circumferences (waist circumference, obtained at the umbilicus level; hip circumference, obtained at the most prominent buttock level); and systolic and diastolic arterial blood pressure (two measurements at 5-minute intervals in seated position; Omron M6; Omron Healthcare Co., Matsusaka, Mie, Japan) were measured according to standardized procedures. Electrocardiogram, carotid echo-Doppler and FMD were obtained for all participants.

### Measurements

#### Carotid intima-media thickness

Images of the right and left extracranial carotid artery walls were obtained in several projections with a high-resolution ultrasonographic 10-MHz linear array probe (Sonoline G50; Siemens, Germany). End-diastolic intima-media thickness (c-IMT) of the far wall of both common carotid arteries was measured as described elsewhere [33]; the highest value was considered for calculations (c-IMTmax). A single physician was responsible for carrying out the carotid ultrasonographic examinations.

#### Assessment of endothelial function

Endothelium-dependent reactivity in the macrocirculation, measured by FMD of the brachial artery, was determined using high-resolution vascular ultrasound (Sonoline G50; Siemens, Germany) with a 10 MHz linear array transducer. The transducer was held at the same position throughout the test by a stereotactic clamp with micrometer adjustment (EDI Progetti e Sviluppo; Pisa, Italy) to ensure image consistency. Reactive hyperemia was produced by inflating a sphygmomanometer cuff 2 cm below the antecubital fossa to occlude the artery for 5 minutes at approximately 220–250 mm Hg, then deflating it. A video processing system computed the brachial artery diameter in real-time by analyzing B-mode ultrasound images (FMD Studio; Institute of Physiology CNR; Pisa, Italy); the device captures the analog video signal from the ultrasound equipment. An edge detection algorithm, based on the localization of gray level discontinuities, automatically locates the two walls of the vessel. The diameter is obtained with subpixel precision and temporal resolution of 25 samples/s. The brachial artery diameters were displayed on a graphic interface over a time scale of 9 minutes. The baseline vessel size was considered the mean of the measures obtained during the first minute. The FMD was calculated as the maximum percentage increase of the brachial artery diameter over baseline. These procedures are described in detail elsewhere [34]–[37]. The FMD test was performed by the same operator, who was blinded to the participant’s classification. Ultrasound images were video recorded and analyzed by a trained reader, who was blinded to the participant’s treatment classification. In our laboratory, the intra-observer coefficient of variation for FMD is 2.9%.

#### Laboratory analysis

Basal lipid measurements, glucose, uric acid, and creatinine were ascertained using standard clinical chemistry methods (Glucosio HK UV; Colesterolo tot. Mod P/D; Colesterolo HDL gen3 mod P/917; Trigliceridi; Acido urico MOD P/917; Creatinina enzimatica; Roche diagnostics, Monza, Italy). Basal insulin concentrations (Elecsys insulina; Roche diagnostics; Monza, Italy) and glycated hemoglobin (HbA_1_c; HbA_1_c gen3; Roche diagnostics; Monza, Italy) were also measured. Low-density lipoprotein (LDL) cholesterol concentration was calculated with Friedewald’s formula [38].

The glomerular filtration rate (GFR) was calculated according to the Modification of Diet in Renal Disease study (MDRD) equation [39]. Insulin resistance was measured in terms of HOMA-IR as described by Matthews et al. [40].

The plasma concentrations of the following metals, which include the transition metals and some metalloids, were measured: mercury, cobalt, lead, copper, zinc, arsenic, selenium, strontium and antimonium. Analysis of metal levels in the blood was done with an inductively coupled plasma mass spectroscopy (ICP-MS Thermo Scientific XSeriesII, Thermo Fisher Scientific, Bremen, Germany). Two ml of blood samples collected in Vacuette K3EDTA tubes were digested using an oxidizing mixture of concentrated nitric acid and hydrogen peroxide with a microwave system (Mars 5; CEM Corporation, Matthews, NC, US). The digested samples were then clarified by filtration, and the concentrations of the metals were determined after dilution with ultrapure water by a factor of 10. An HNO_3_ blank was run during the analysis to ensure that the memory effect, due to the more refractory elements, was negligible.

### Statistical Analysis

An expected difference in FMD between the two groups was estimated to be at least 3%. The power analysis showed that with an α error of 0.05, a pooled standard deviation of 2.5, and a power of 0.80, 12 participants were needed per group.

All data are presented as mean ± standard deviation (SD) or as prevalence (%). Comparisons between groups were tested for statistical significance with the unpaired Student’s t-test or Fisher’s χ^2^, when appropriate. Linear regression analysis assessed the relationships between variables. A two-tailed P<0.05 was considered significant. All analyses were performed with Systat (Windows version 11.0; San Jose, CA, USA).

## Results

**Figure 1:**
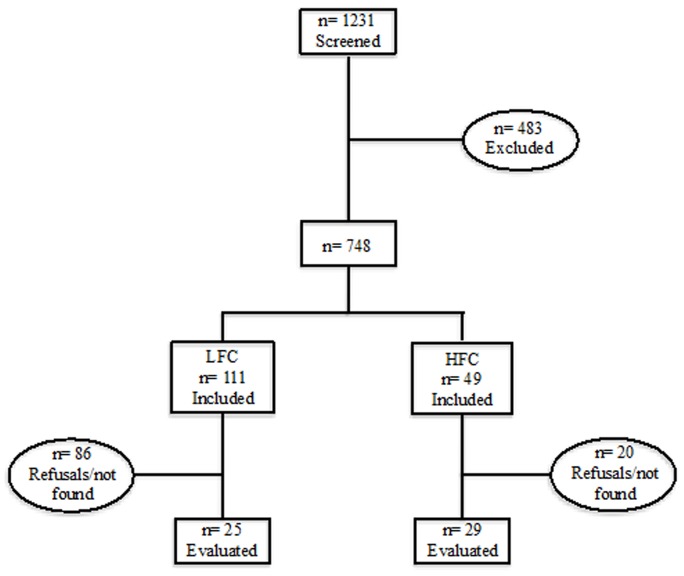
Participant Selection Flow (LFC = Low Habitual Fish Consumers; <1 Portion a Week. HFC = High Fish Consumers; ≥3 Portions a Week).

**Figure 2:**
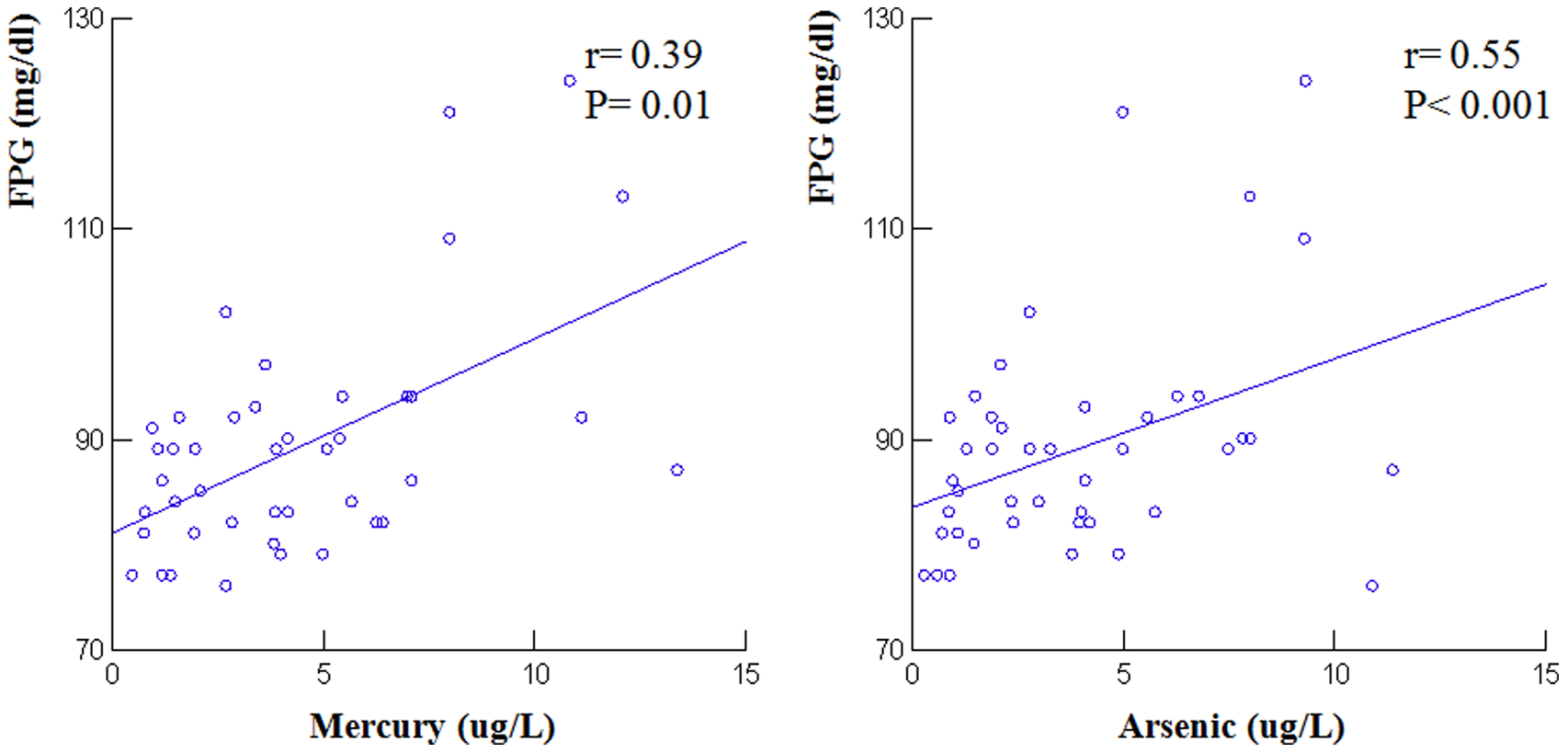
Correlations between Fasting Plasma Glucose (FPG) and Both Mercury and Arsenic Serum Concentrations.

A total of 54 participants were evaluated: 29 HFC and 25 LFC (Figure 1). Serum concentrations of metals and metalloids could be measured in 24 HFC and in 19 LFC participants. The characteristics of the two groups are presented in Table 1. In terms of dietary patterns, the LFC group had a lower prevalence of participants who followed the Mediterranean diet, and a higher prevalence of Unhealthy dietary pattern compared with the HFC group. However, differences were not significantly different between the two groups (P = 0.06). FMD was significantly higher in the HFC group than in the LFC group (9.7±1.8 vs. 7.3±1.9%; P<0.001). The HFC group had also higher blood concentrations of both mercury (5.87±2.69 vs. 1.65±1.10; P<0.001) and arsenic (6.04±3.25 vs. 2.30±1.58; P<0.001) than the LFC group. No significant difference between the two groups was observed concerning anthropometric and other clinical and laboratory data (Tables 2 and 3). The FPG concentrations were significantly correlated with the blood concentration of both mercury (r = 0.39; P = 0.01) and arsenic (r = 0.55; P<0.001) (Figure 2). No other significant correlation between metal concentration and the variables considered in the study were observed.

**Table 1:** Demographic and Clinical Characteristics of the Cohort Categorized according to the Level of Habitual Fish Consumption.

	Fish consumers		
	High(n = 29)	Low(n = 25)	P^1^
Age (y)	54±12	50±12	0.22
Gender (male/female)	13/16	7/18	0.20
Smokers, n (%)	9 (16.7)	11(20.4)	0.15
Number of Offspring	1.5±1.3	1.6±1.0	0.85
Prevalence of hypertension, n (%)	6 (11.1)	9 (16.7)	0.21
Use of anti-hypertensives, n (%)			
diuretics	1 (1.9)	2 (3.7)	0.47
beta-blockers	3 (5.6)	1 (1.9)	0.38
ACEI or ARBs	1 (1.9)	4 (7.4)	0.11
Dietary pattern, n (%)			0.06
Mediterranean	12 (22.2)	7 (12.9)	
Intermediate	15 (27.8)	10 (18.5)	
Unhealthy	2 (3.7)	8 (14.8)	
Fish consumption (portions/week)	4.4±1.5	0.04±0.2	<0.001

All values are presented as means ± SD or in absolute values (percentage in parenthesis).

1 Student’s unpaired t-test or Fisher’s χ^2^, when appropriate.

ACEI: angiotensin converting enzyme inhibitors. ARBs: angiotensin receptor blockers.

**Table 2:** Anthropometric, Echographic and Laboratory Data of the Cohort Categorized according to the Level of Habitual Fish Consumption.

	Fish consumers		
	High(n = 29)	Low(n = 25)	P^1^
Body weight (kg)	70.4±10.7	72.9±15.1	0.50
BMI (kg/m^2^)	26.6±4.4	26.7±3.9	0.91
Waist circumference (cm)	92±10	92±12	0.78
Waist-to-hip ratio	0.89±0.07	0.88±0.08	0.93
Blood pressure (mmHg)			
systolic	131±16	127±11	0.26
diastolic	76±9	79±7	0.20
Heart rate (beats/min)	75±13	73±9	0.48
Carotid-IMTmax (mm)	0.61±0.12	0.62±0.17	0.80
FMD (%)	9.7±1.8	7.3±1.9	<0.001
Blood concentration of:			
glucose (mg/dL)	92±16	91±13	0.57
glycated hemoglobin (%)	5.4±0.6	5.4±0.5	0.87
insulin (µU/ml)	7.8±4.8	9.3±4.9	0.26
total cholesterol (mg/dl)	226±34	211±32	0.11
HDL-cholesterol (mg/dl)	67±20	63±17	0.39
triglycerides (mg/dl)	88±40	99±47	0.37
LDL-cholesterol (mg/dl)	144±31	129±28	0.06
uric acid (mg/dl)	5.0±1.4	4.4±1.0	0.10
creatinine (mg/dl)	0.81±0.18	0.78±0.13	0.53
GFR - MDRD (mL/min/1.73 m^2^)	106.3±12.9	103.7±11.6	0.44
HOMA-IR	1.95±1.78	2.64±2.38	0.24

All values are presented as means ± SD.

1 Student’s unpaired t-test.

FMD: flow-mediated dilation of the brachial artery; GFR: glomerular filtration rate; HDL: high-density lipoproteins; MDRD: Modification of Diet in Renal Disease study; LDL: low-density lipoproteins.

**Table 3:** Serum Concentrations of Metals, Including Some Transition Metals and Metalloids, in the Cohort Categorized according to the Level of Habitual Fish Consumption.

	Fish consumers		
	High(n = 24)	Low(n = 19)	P^1^
Mercury (202) ug/L	5.87±2.69	1.65±1.09	<0.001
Cobalt (59) ug/L	0.74±0.18	0.65±0.19	0.12
Lead (208) ug/L	25.4±12.4	26.6±14.2	0.77
Copper (63) ug/L	511±202	694±184	0.17
Zinc (66) ug/L	4618±261	4784±314	0.07
Arsenic (75) ug/L	6.04±3.25	2.30±1.58	<0.001
Selenium (78) ug/L	158.3±54.5	141.8±44.2	0.29
Strontium (88) ug/L	24.1±9.8	22.5±6.3	0.54
Antimonium (121) ug/L	4.07±0.88	3.53±1.01	0.07

All values are presented as means ± SD. Atomic weight in brackets.

1 Student’s unpaired t-test.

## Discussion

We investigated two groups of apparently healthy people with similar anthropometric, nutritional and clinical characteristics, but quite different habitual fish consumption. In particular, we had no clinical evidence of a different atherosclerotic involvement between the two groups. Levels of c-IMTmax, a marker of subclinical atherosclerosis, were similar. Therefore, even in people without established atherosclerosis, we found that consuming fish at least 3 times a week was associated with better endothelial function, measured in terms of FMD, than was consuming fish less than once a week. These results are in agreement with studies that found an inverse association between habitual fish intake and markers of inflammation or endothelial dysfunction [20], [41]. Few studies have investigated endothelial function in frequent fish eaters and in people who habitually do not eat fish. In contrast with our results, Petersen et al. did not find any significant difference in FMD comparing a group of people who habitually consumed at least 300 g of fish per week (corresponding to the Danish recommendations) with a group that habitually did not consume fish [42]. A possible explanation is that the effect of fish intake on FMD is significant for habitual intake of high amounts of fish. In fact, the intake of fish of our HFC participants was on average 4.4 portions/week, and assuming that a portion of fish is about 150–200 g, their weekly intake of fish was at least of 600–800 g/week, which is well above the Danish recommendations. Furthermore, the cardiovascular health benefits of fish are due, at least in part, to the n-3 PUFAs present in many species. Even in this case, when the effect of low doses of n-3 PUFAs on FMD was tested in healthy adults no significant improvement was observed [43]. On the contrary, higher amounts of n-3 PUFA consumption sharply improved FMD following a saturated-fatty-acid-rich drink [44]. Similarly, the effect of fish oil supplementation on FMD has not been firmly established [21], and a study by Plat et al. failed to demonstrate beneficial effects of fish oil on biomarkers of endothelial function and inflammation [45]. However, n-3 PUFAs are only one of the components of fish that may contribute to its favorable cardiovascular effects, including those on FMD. Vitamins A, B and D, calcium, phosphorus, iron, copper and selenium should in fact be considered, though we did not investigate them, with the exception of selenium, which had blood concentrations slightly but not significantly higher in the HFC group. Indeed, consumption of fish as a source of n-3 PUFAs has been found to be superior to the use of fish oil supplements, making fish intake the most effective source of n-3 PUFAs [46]. Our study reinforces the notion that fish is an important component of a healthy diet, with protective cardiovascular effects that are mediated, at least in part, by beneficial effects on the endothelium. Beyond its beneficial effects on coronary artery disease [47], [48], fish consumption has also been associated with reduced risk of ischemic stroke [49]–[52] and, interestingly, it has been suggested that racial and geographic differences in fish consumption may help explain racial and geographic differences observed in stroke incidence and mortality [53].

The favorable cardiovascular effects of consuming significant amounts of fish should, however, be balanced with the possible risks of exposure to contaminants, mainly metals. The presence of select toxic metals has been reported even in fish from Sicily, the largest island in the Mediterranean Sea, which was the site of our study [54], [55]. This is likely why we found significantly higher blood concentrations of mercury and arsenic in the HFC group. Studies that measured the toxic metal content of fish have generally concluded that despite the fact that they are significantly present, their concentrations are well below those indicated as having toxic effects on human health [54], [56]–[58]. It is perhaps appropriate to relate risk of exposure to toxic metals, including the transition metals and some metalloids, to the amount and frequency of habitual fish intake. We found that the serum concentrations of mercury and arsenic were significantly higher in HFC participants than in non-consumers, but we do not know if a different health risk corresponds to such differences in blood concentrations. Based on data from Moon et al., in a South Korean population, the average blood concentrations of mercury that we observed in the HFC group correspond to the fourth quartile of distribution in the Moon study, and should therefore be considered elevated [59]. Mercury may increase the risk of myocardial infarction [23], and may induce oxidative stress [60]. Our short term study indicates that despite higher serum mercury and arsenic concentrations, improved endothelial function was observed in the HFC group. Our study therefore seems to agree with other reports [61] that the cardiovascular benefits of fish intake exceed the potential risks. However, longer follow-up would be required to determine whether increased levels of metal contaminants have negative effects on health. There are, for example, reports suggesting that mercury and arsenic exposure increase the risk of diabetes [27], [28]. Our finding of a significant correlation between fasting plasma glucose and both mercury and arsenic concentrations supports this idea.

Our study has important limitations. We did not obtain metal measurements in all study participants, and we measured concentrations of metals in serum and not in hair or erythrocytes, as usually required when exposure to these substances needs to be investigated. However, the choice of measuring metals in serum was based on the availability of samples, and it has been demonstrated that serum Hg concentration is also a reasonably good index of exposure to mercury [62]. In addition, serum concentrations may be related to exposure to metals in the short term, compared with measurements in hair and nails, which reflect longer exposures. This may explain why we found significant correlations between FPG and mercury and arsenic serum concentrations, while we found no significant correlations with other measures of glucose control, such as glycated hemoglobin, which reflect glucose levels for the preceding 3 months.

Habitual fish intake may, generally speaking, reflect healthier dietary habits, which are responsible for the favorable effects on the endothelial function that we found associated with fish consumption. However, in this study we reported the prevalence of dietary patterns as obtained in the complete cohort we presented elsewhere [31]. The HFC group exhibited healthier dietary habits, but differences with LFC group did not reach statistical significance. Given the cross-sectional design of the study, we cannot exclude the possibility of residual confounding factors. Also, we cannot determine whether differences in the favorable endothelial effects of fish consumption exist according to differences in fish cooking-procedures (fried or not) or in its provenance (farm-raised fish or their wild counterparts), storage (frozen or fresh) or typology (fatty or lean fish).

The strengths of this study are the modality of participant recruitment, which allowed for the characterization of study participants, and the use of a strict ultrasound procedure for FMD measurement by one operator, which may have contributed to reducing possible biases.

In conclusion, despite higher levels of toxic metals such as mercury and arsenic, we observed a favorable association between fish consumption and endothelial function in apparently healthy people. Though these results may encourage the consumption of fish, intervention studies in the medium to long term are needed to test the effects of this food on metabolic and cardiovascular end-points.
